# Including Protons in Solid-State NMR Resonance Assignment and Secondary Structure Analysis: The Example of RNA Polymerase II Subunits Rpo4/7

**DOI:** 10.3389/fmolb.2019.00100

**Published:** 2019-10-04

**Authors:** Anahit Torosyan, Thomas Wiegand, Maarten Schledorn, Daniel Klose, Peter Güntert, Anja Böckmann, Beat H. Meier

**Affiliations:** ^1^Physical Chemistry, Eidgenössische Technische Hochschule Zurich, Zurich, Switzerland; ^2^Center for Biomolecular Magnetic Resonance, Institute of Biophysical Chemistry, Goethe University Frankfurt, Frankfurt, Germany; ^3^Department of Chemistry, Tokyo Metropolitan University, Hachioji, Japan; ^4^Institut de Biologie et Chimie des Protéines, MMSB, Labex Ecofect, UMR 5086 CNRS, Université de Lyon, Lyon, France

**Keywords:** Rpo4/7, solid-state NMR, carbon and proton assignments, secondary chemical shifts, ssFLYA

## Abstract

^1^H-detected solid-state NMR experiments feasible at fast magic-angle spinning (MAS) frequencies allow accessing ^1^H chemical shifts of proteins in solids, which enables their interpretation in terms of secondary structure. Here we present ^1^H and ^13^C-detected NMR spectra of the RNA polymerase subunit Rpo7 in complex with unlabeled Rpo4 and use the ^13^C, ^15^N, and ^1^H chemical-shift values deduced from them to study the secondary structure of the protein in comparison to a known crystal structure. We applied the automated resonance assignment approach FLYA including ^1^H-detected solid-state NMR spectra and show its success in comparison to manual spectral assignment. Our results show that reasonably reliable secondary-structure information can be obtained from ^1^H secondary chemical shifts (SCS) alone by using the sum of ^1^H^α^ and ^1^H^N^ SCS rather than by TALOS. The confidence, especially at the boundaries of the observed secondary structure elements, is found to increase when evaluating ^13^C chemical shifts, here either by using TALOS or in terms of ^13^C SCS.

## Introduction

Solid-state NMR and, in particular, proton-detected spectroscopy under fast MAS allows to characterize larger and larger proteins and protein complexes (Linser et al., 2011; Andreas et al., 2015; Struppe et al., 2017; Schubeis et al., 2018; Bougault et al., 2019). Here, we demonstrate the resonance assignment and secondary-structure determination of the subunit Rpo7 of the archaeal DNA-dependent RNA polymerase (RNAP) in the context of the protein complex Rpo4/Rpo7 (33.5 kDa). RNAPs from bacteria, archaea, and eukarya are well-characterized in terms of their subunit composition, as well as their structure, and much is known about the regulation mechanisms and complex interplay of transcription factors throughout the transcription cycle of initiation, elongation, and transcription termination (Werner and Grohmann, 2011; Sainsbury et al., 2015; Hantsche and Cramer, 2016). Especially the archaeal RNAP has served as a model system for dissecting the functions of the individual subunits of the human RNAP II (Werner, 2007, 2008).

Two of these subunits, Rpb4/Rpb7, that form a stalk-like protrusion in RNAP II, or rather their archaeal homologs Rpo4/Rpo7 (or Rpo4/7), are known to bind the nascent single-stranded RNA, contribute to transcription initiation as well as termination efficiency and increase processivity during elongation (Meka, 2005; Újvári and Luse, 2006; Grohmann and Werner, 2010, 2011). Yet, how these functions are achieved in molecular detail remains elusive, and conformational changes of Rpo4/7 in response to RNA binding have not been detected when probed by labeling techniques, such as fluorescence and electron paramagnetic resonance spectroscopy (Grohmann et al., 2010). NMR spectroscopy could provide further information at the atomic level.

As a first step, we present the ^1^H, ^13^C, and ^15^N protein resonance assignment employing solid-state MAS experiments of a sedimented Rpo4/7 complex from the archeon *Methanocaldococcus jannaschii*. For this, we labeled the Rpo7 subunit uniformly with ^13^C/^15^N, while Rpo4 was employed at natural isotopic abundance. This enabled us to selectively study the Rpo7 subunit within the complex. We assigned, on the basis of the acquired spectra and using different assignment strategies, ~80% of the C^α^, C^β^, and backbone nitrogen atoms. It has been demonstrated that NMR chemical-shift values encode for the secondary structure (Wishart et al., 1992; Wishart and Sykes, 1994; Wang, 2002; Shen et al., 2009). We compared the secondary structure predictions based on the different chemical shifts, and compared them also to the known crystal structure. We found that for proton resonances, the most reliable information can be derived from ^1^H secondary chemical shifts (SCS) using the sum of ^1^H^α^ and ^1^H^N^ SCS. Nevertheless, ^13^C chemical shifts are found to be more reliable in terms of secondary-structure information, both directly from SCS and from TALOS.

## Materials and Methods

### Protein Expression and Purification, Sample Preparation

Plasmids pET21_Rpo7 and gGEX_2k_Rpo4 were transformed into *E. coli* BL21 (DE3) cells separately for Rpo4 and Rpo7. Rpo4 was overexpressed with an N-terminal glutathione S-transferase (GST)-tag in rich medium (Terrific Broth, 2006) and purified via affinity chromatography using glutathione agarose (GSTrap, GE Healthcare, Glattbrugg, Switzerland) using P100 buffer (20 mM tris/acetate pH 7.9, 100 mM K acetate, 10 mM Mg acetate, 0.1 mM ZnSO_4_, 5 mM DTT, 10% (w/v) glycerol) and 10 mM reduced glutathione for elution, similar to previous protocols (Werner and Weinzierl, 2002; Klose et al., 2012). The GST-tag was cleaved by overnight incubation with thrombin at 37°C. To deactivate and remove the GST-tag, a 20-min heat shock of the cleaved elution fractions at 65°C was applied with subsequent centrifugation (13,000 rpm, 20 min, 4°C), leaving purified Rpo4 in the supernatant. For isotope labeling with ^15^N and ^13^C, Rpo7 mutant S65C was expressed in M9-minimal medium (Studier, 2005) consisting of 6.8 g Na_2_HPO_4_, 3 g KH_2_PO_4_, 0.5 g NaCl, 1 ml of each 1 M MgSO_4_, 10 mM ZnCl_2_, 1 mM FeCl_3_, and 100 mM CaCl_2_ per 1 L medium, supplemented with 10 ml MEM vitamin solution (100×). One gram ^15^NH_4_Cl and 2.5 g ^13^C-glucose (Cambridge Isotope Laboratories, Tewksbury, USA) were the only nitrogen and carbon sources. Rpo7^*^ (the asterisk denotes isotope labeling) purification from inclusion bodies was carried out as described previously (Werner and Weinzierl, 2002; Klose et al., 2012).

The complex formation of Rpo4 and Rpo7^*^ (with 20% excess) was carried out by unfolding and stepwise refolding dialysis in P100 buffer using urea (6, 4, 3, 2, 1, 0.5, and 0 M urea concentrations, 1 h per step, room temperature). Subsequently, a 20 min heat shock at 65°C and a subsequent centrifugation step (8,000 × g, 20 min, 4°C) was applied to remove excess or misfolded Rpo7^*^ after the dialysis. Purity and stability of the complex was confirmed by SDS and native page (Figure S1). All chemicals were of p.a. grade and purchased from Sigma Aldrich (Buchs, Switzerland), unless stated otherwise.

### Solid-State NMR Spectroscopy

Rpo4/7^*^ supplemented with DSS and sodium azide was sedimented into NMR rotors (0.7 and 3.2 mm, Bruker Biospin, Rheinstetten, Germany) by ultracentrifugation (35,000 rpm, 4°C, 16 h) using home-made filling tools (Böckmann et al., 2009) resulting in 0.6 and 24 mg protein in the rotors with 0.7 and 3.2 mm diameter, respectively. Solid-state NMR spectra were recorded on a Bruker AVANCE III 850 MHz NMR spectrometer using either a 3.2 mm Bruker “E-free” probe or a 0.7 mm Bruker triple-resonance probe. The MAS spinning frequencies were set to 17.0 kHz for the 3.2 mm rotor and 110 kHz for the 0.7 mm rotor, with sample temperatures of 16°C (lowest possible temperature in this set-up) and 5°C for the 0.7 and 3.2 mm rotors, respectively. The 2D and 3D spectra were processed with TopSpin (version 3.5, Bruker Biospin, Rheinstetten, Germany) and analyzed in CcpNmr Analysis 2.4.2 (Stevens et al., 2011). More details of the conducted experiments are presented in Table S1. Polarization transfers between H-C and H-N used adiabatic cross polarization (Hediger et al., 1995), as did N-C polarization transfers (Baldus et al., 1996), while C-C transfers used either DARR (Takegoshi et al., 2003) or DREAM (Verel et al., 2001).

The ^13^C-detected spectra used for the assignment were all recorded on a single sample (3.2 mm rotor). Reproducibility was checked by 2D measurements on samples from two different preparations in 0.7 mm rotors, which yielded identical spectra in all cases.

The obtained assignment was deposited in the BioMagResBank under accession number 27959.

### TALOS+ Predictions and FLYA Calculations

TALOS+ predictions were performed using version 3.8 (Shen et al., 2009). The secondary structure assignments based on the DSSP algorithm (Kabsch and Sander, 1983) were used as given in the corresponding PDB entry 1GO3 (Todone et al., 2001) and the 3D atomic coordinates were extracted from the same PDB entry. Solid-state FLYA calculations (Schmidt and Güntert, 2012; Schmidt et al., 2013) were performed with CYANA version 3.97 (Güntert and Buchner, 2015). Peak lists of ^13^C and ^1^H-detected spectra were used, using the peak lists from the resonance assignment (manual peak lists) or using automatically generated peak lists. Automated peak picking has been performed in CcpNmr using the implemented picking routine. The lowest contour level was set to 2.0–3.0 time noise RMSD for this process. The tolerance value for chemical-shift matching was set to 0.55 ppm for ^13^C, ^15^N, and 0.3 ppm for ^1^H.

## Results and Discussion

### Assignment of ^13^C Detected Solid-State NMR Spectra

**Figure 1:**
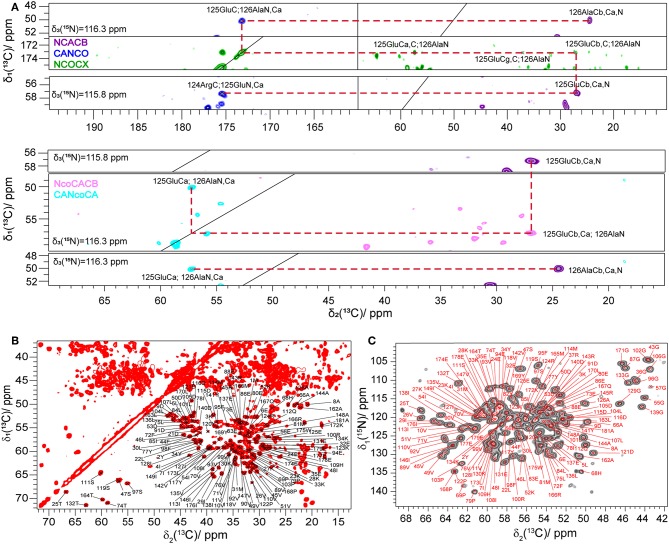
**(A)** Example of a ^13^C, ^15^N sequential resonance walk. **(B)** 2D ^13^C, ^13^C DARR spectrum of Rpo4/7^*^ measured at 20.0 T with a MAS frequency of 17 kHz and a DARR mixing time of 20 ms. **(C)** 2D NCA spectrum of Rpo4/7^*^ measured at 20.0 T with a MAS frequency of 17 kHz. In **(B,C)**, C^α^, and C^β^ peaks are labeled according to the manually created shift list using the CcpNmr software.

The ^13^C and ^15^N-MAS solid-state NMR spectra of Rpo4/7^*^ show well-dispersed signals and roughly the expected number of peaks (Figure S2) in the region of serine (four out of six expected peaks), threonine (4/4), alanine (7/8), and glycine (12/16) as can be seen in the 2D dipolar correlation spectra in Figure 1, suggesting that the sample contains Rpo4/7^*^ in a single, well-defined conformation. The ^13^C-linewidths are on the order of 115 Hz, which points to a homogeneous sample.

Seven 3D ^13^C-detected spectra (NCACB, NCACX, CANCO, NCOCX, NcoCACB, CANcoCA, and CCC) were measured to obtain the ^13^C and ^15^N assignment. The ^13^C and ^15^N assignment was mainly achieved by a combination of two strategies described earlier (Schuetz et al., 2010) and shown in Figure 1A. The first is based on a sequential walk using NCACB, CANCO, NCOCX, the second uses the relayed experiments NcoCACB and CANcoCA, in combination with NCACB. The side chains were mainly assigned by analyzing NCACX and CCC spectra [employing Dipolar Recoupling Enhanced by Amplitude Modulation (DREAM) (Verel et al., 2001; Westfeld et al., 2012) and Dipolar Assisted Rotational Resonance (DARR) (Takegoshi et al., 2003) transfer steps].

**Figure 2:**
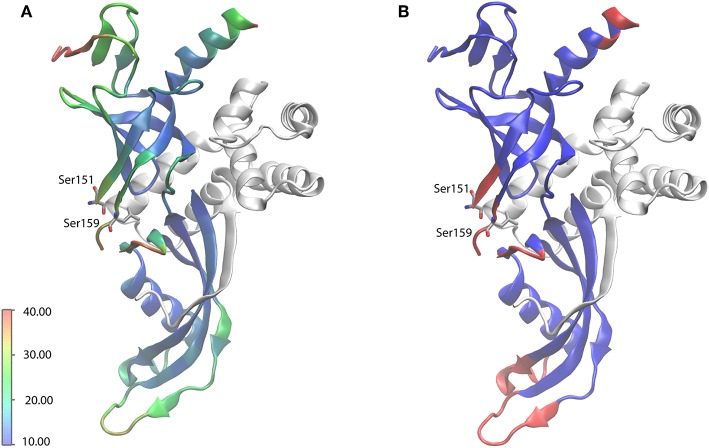
X-ray crystal structure of Rpo4/7 (PDB: 1GO3). Rpo4 is shown as white ribbons. **(A)** Rpo7 (ribbons), colored according to the crystallographic *B* factor (see scale bar, in Å^2^). **(B)** Rpo7 (ribbons), colored blue and red for backbone-assigned and unassigned residues, respectively. The RNA-binding loop, the region with the highest flexibility, for which no coordinates are available, is indicated by the flanking residues S151 and S159.

Manual analysis of all 3D spectra resulted in the assignment shown in the 2D ^13^C, ^13^C DARR (Figure 1B) and 2D ^15^N, ^13^C NCA (Figure 1C) spectra, where 99% of all visible peaks are assigned. The assignment graph is shown in Figure S3. Statistics of the manually performed peak assignment is shown in Table S2. The resonances of most of the unassigned residues could thus neither be detected in 3D nor in 2D spectra, most probably because they are located in flexible parts of the protein. Figure 2 illustrates the spatial correlation between unassigned residues and the crystallographic *B*-factor, which shows that the most flexible part, the RNA binding loop (Meka, 2005), which is not resolved in the crystal structure (Todone et al., 2001), is found to be close to the unassigned residues Ser151–Ser159. The invisible residues are, however, not flexible enough to be visible in an INEPT spectrum (data not shown).

### Assignment of ^1^H-detected Solid-State NMR Spectra

**Figure 3:**
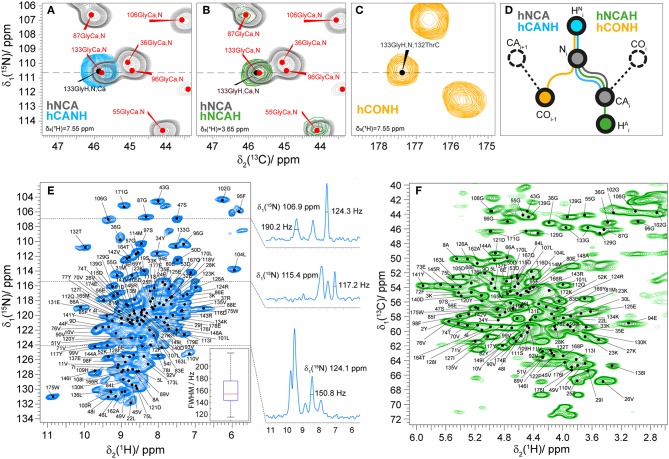
**(A)** 2D NCA spectrum (gray) and 2D plane of a 3D hCANH (cyan) spectrum at δ(^1^H) = 7.6 ppm showing an example of the assignment transfer for 133Gly; **(B)** 2D NCA (gray) spectrum and 2D plane of a 3D hNCAH (blue) spectra at δ(^1^H) = 3.7 ppm showing the example of the assignment transfer for 133Gly; **(C)** 2D plane of hCONH spectrum δ(^1^H) = 7.6 ppm; **(D)** schematic representation of the assignment transfer for H^N^ and H^α^ atoms; **(E)** 2D hNH correlation spectrum of fully protonated Rpo4/7^*^ at 110 kHz MAS. The spectrum includes labels for the ^15^N-^1^H peaks as predicted from the manually created shift list. On the right side of the figure 1D traces for ^1^H are presented at the corresponding ^15^N frequencies. The ^1^H linewidth characteristics of the full population of marked cross-peaks are summarized in the boxplot in the bottom right, indicating the maximum, 3rd quartile, mean, 1st quartile and minimum value of proton FWHM linewidth in Hz with a mean value of 160 ± 40 Hz. **(F)** 2D hCH correlation spectrum of fully protonated Rpo4/7^*^ at 110 kHz MAS with peaks labeled as in **(E)**.

To assign the amide H^N^ and aliphatic H^α^ protons of fully protonated Rpo7^*^ in complex with Rpo4, we used proton-detected spectroscopy at 110 kHz MAS frequency. The assignment of the 2D hNH fingerprint spectrum is shown in Figure 3. The assignment was done using three 3D spectra, namely hCANH, hNCAH, and hCONH (Barbet-Massin et al., 2014; Penzel et al., 2015), and taking advantage of the ^13^C and ^15^N peak assignment described above. Details of the experiments are given in Table S1. The assignment of the NCA spectrum was transferred peak by peak to hCANH (Figures 3A,D) and hNCAH (Figures 3B,D) spectra. To confirm the assignment of amide protons, an additional hCONH spectrum was used to verify the CO chemical shift of the previous residue (Figures 3C,D). In total, 97% of the amide protons and 93% of the H^α^ protons for which C^α^ and N assignments exist could be assigned. In the assignment graph of Figure S2 those atoms are highlighted in blue and red, respectively.

The mean value and standard deviation of the ^1^H linewidths of the fully protonated hNH spectrum are 156 ± 40 Hz for all the peaks marked in Figure 3E. On the right side of the spectra 1D traces of ^1^H are shown at the corresponding ^15^N frequencies with linewidths of selected peaks.

The results of the manual assignment procedure were validated by automated resonance assignments as implemented in the solid-state FLYA algorithm (Schmidt and Güntert, 2012; Schmidt et al., 2013). In addition to the ^13^C and ^15^N chemical shifts, ^1^H solid-state chemical shifts were assigned as well in an automated process. Figure S4A illustrates the good agreement between the manual assignments and the assignments obtained by FLYA. For residues shown in green, the FLYA assignment agreed with the manual assignment (within a tolerance of 0.55 ppm for ^13^C, ^15^N, and 0.3 ppm for ^1^H). A few significant differences (red) were observed. In those cases, the manual assignment was carefully verified and found to be consistent. Agreement (including both dark and light green residues) between FLYA and the manually assigned backbone atoms was found for 95% of ^15^N, 92% of ^13^C', 95% of ^13^C^α^, 87% of H^N^, and 89% of H^α^ atoms. The FLYA algorithm was also applied using automatically picked peak lists as input, and we found agreement to 82% of ^15^N, 84% of ^13^C', 82% of ^13^C^α^, 75% of H^N^, and 76% of H^α^ atoms (Figure S4B). We conclude that the automatic assignment provides a good starting point for manual assignment or a good check of manual results.

### Secondary Structure From ^13^C- and ^1^H-detected Spectra

**Figure 4:**
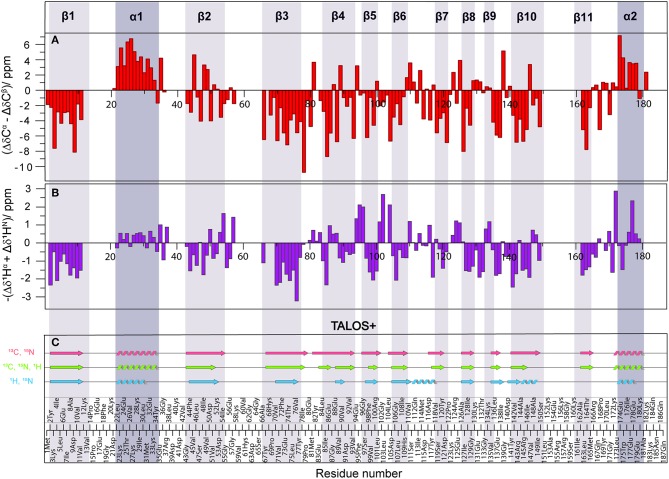
**(A)** Difference of Δδ(^13^Cα) and Δδ(^13^Cβ) secondary chemical shifts (SCS) (red). **(B)** Negative sum of ^1^H^α^ and ^1^H^N^ SCS (purple). SCS are obtained by subtracting the random-coil shifts from the observed chemical shifts. Positive SCS differences indicate α-helices, negative SCS difference β-sheets. **(C)** Secondary structure based on ^13^C and ^15^N (light red), ^1^H and ^15^N (light blue) and all (light green) chemical shifts using TALOS+ (Shen et al., 2009). Secondary structure elements observed by crystallography are shown as dark (α-helix) and light (β-sheet) gray shaded areas, according to PDB 1GO3 (Todone et al., 2001).

In order to compare the secondary structure determined by different approaches from solid-state NMR chemical shifts, either using SCS or by backbone dihedral angle statistics [TALOS+ (Shen et al., 2009)], we used the X-ray crystal structure of Rpo4/7 determined at 1.75 Å [PDB: 1GO3 (Todone et al., 2001)] as a common reference. The positions of the secondary structure elements were determined from the X-ray coordinates via the algorithm DSSP (Kabsch and Sander, 1983). The results are indicated at the top of Figure 4, Figures S5, S6 as well as by the gray bars.

As an indicator for the secondary structure, the SCS of C^α^, C^β^, CO, as well the SCS difference of C^α^ and C^β^ were calculated and are visualized in Figures S5, S6. For solid-state NMR, the most commonly used indicator is ΔδC^α^-ΔδC^β^ which has the advantage of being independent from reference errors (Spera and Bax, 1991). Three or more negative values in a row indicate a β-sheet, four or more positive values an α-helix. For reference, the positions of the secondary structure elements were determined from the X-ray coordinates. The results are indicated in Figure 4A, Figures S5A, S6A, and Table S3. Overall, the correspondence is good, with some significant deviations in the β-strands, in particular β2. Upon visual inspection of the structure of β2 and β3 in the crystal structure (Figure S7), it becomes clear that this is related to the fact that β2 is rather distorted and irregular, while β3 is more regular. The difference between these two β-sheets is also clearly seen in the Ramachandran plots (Figure S8). The differences in the NMR SCS are therefore based on actual structural properties.

To obtain secondary-structure information from proton-detected fingerprint spectra, SCS of both ^1^H^α^ and ^1^H^N^ were used (Figure 4B, Figures S5B, S6A, Table S3). It is well-known (Wang, 2002), that ^15^N SCS is a poor indicator for secondary structure (Figures S5, S6, orange). Instead, the sum of ^1^H^α^ and ^1^H^N^ SCS appears to be a suitable measure for secondary structure identification (Figure 4, Figures S5, S6, purple), even though summing up doesn't compensate for referencing errors. While not as precise as the ^13^C chemical shifts, the sum of the two proton SCS still provides useful information about secondary structure.

Our results are similar to solution NMR in that SCS data of ^1^H^α^ for α-helices were found more reliable than that of ^1^H^N^ (Wang, 2002). We found the ^13^C^α^-^13^C^β^ SCS data to be a more suitable indicator than SCS sum ^1^H^α^ + ^1^H^N^ data. Similarly, ^1^H^α^ SCS were shown (Wang, 2002) to be on average more sensitive in distinguishing β-sheets from random coil conformations than ^13^C^α^ and ^13^C^β^ chemical shifts. In our case ^13^C^α^-^13^C^β^ SCS data were the most reliable. However, for big proteins where transfer efficiencies are not always good, ^13^C^β^ data may be unavailable (Penzel et al., 2015; Stöppler et al., 2018). We identified that, besides of ^13^C^α^ SCS, the sum of ^1^H^α^ and ^1^H^N^ SCS is a suitable alternative parameter to derive secondary structure.

Additionally, secondary-structure elements were predicted using the software TALOS+ (Shen et al., 2009) and are shown in Figure 4C. Three different combinations of chemical shifts derived from manual assignment were used: ^13^C and ^15^N, ^1^H and ^15^N, and all three available shifts. The combination of ^13^C and ^15^N data extracted using TALOS+ (light red) yielded the most promising results, as the predicted secondary structure fits well with the crystal structure, including strand β2 and β10 that were only incompletely recognized by the SCS data. Surprisingly, TALOS+ results did not improve upon inclusion of ^1^H chemical shifts (light green); instead a disruption for strand β4 appeared and strands β2, β5, and β10 became shorter (see also Figure S9 for a comparison in terms of backbone dihedrals). In order to check the reliability of TALOS+ secondary structure results for cases where ^13^C data are absent, we evaluated the combination of ^1^H and ^15^N chemical-shift values (light blue). The calculation resulted in two additional misplaced α-helices, which was not the case for other chemical-shift combinations that included ^13^C data. Therefore, while TALOS+ predictions that included ^13^C chemical shifts were successful, calculations including only ^1^H and ^15^N chemical shifts were here found to be less reliable than SCS analysis when the sum of ^1^H^α^ and ^1^H^N^ SCS is used.

## Conclusions

Using MAS solid-state NMR, we sequentially assigned 78% of the ^13^C, ^15^N resonances of the RNA polymerase subunit Rpo7 in complex with unlabeled Rpo4, and successfully transferred these to ^1^H detected NMR spectra assigning ~70% of the ^1^H^N^ and ^1^H^α^ resonances. Further assessing the secondary structure in comparison to the known crystal structure, our results confirm that ^13^C SCS are a *bona fide* predictor of secondary structure elements. While using only ^1^H^α^ or ^1^H^N^ SCS alone showed an increased uncertainty in the boundaries of observed secondary structure elements compared to the crystal structure, in cases where ^13^C^β^ chemical shifts are not available, secondary structure elements can be identified using either ^13^C^α^ or the sum of ^1^H^α^ and ^1^H^N^ SCS.

The proton assignment forms the basis for protein-nucleic acid interaction studies to identify the RNA-binding sites of Rpo4/7 through ^1^H chemical-shift perturbations. Proton chemical-shift values are in particular sensitive to non-covalent interactions involved in molecular recognition and thus serve as sensitive reporters. Also, the investigation of the molecular dynamics becomes accessible, in the presence and absence of nucleotides, through ^15^N *R*_1ρ_ and *R*_2_' relaxation-rate constants that, once protons are assigned, are measured most efficiently in a series of hNH fingerprint spectra or, with higher resolution, in hCANH spectra.

## Data Availability Statement

All datasets generated for this study are included in the manuscript/Supplementary Files.

## Author Contributions

AT carried out protein syntheses and analyses, and generated NMR samples with support of DK. AT, with the help of TW and MS, conducted the NMR experiments and analyzed the data. PG extended FLYA capabilities and supported FLYA calculations carried out by TW. AT wrote the manuscript with input from all authors. TW, AB, and BM designed and supervised the study. All authors approved the submitted version.

### Conflict of Interest

The authors declare that the research was conducted in the absence of any commercial or financial relationships that could be construed as a potential conflict of interest.
