# Supplementary material for: Including Protons in Solid-State NMR Resonance Assignment and Secondary Structure Analysis: The Example of RNA Polymerase II Subunits Rpo4/7
Source: Front Mol Biosci. 2019 Oct 4;6:100. doi: 10.3389/fmolb.2019.00100 (PMC6787281; doi:10.3389/fmolb.2019.00100)
Supplement: Supplementary file 1 [file Data_Sheet_1.PDF]

Including protons in solid-state NMR resonance assignment and secondary structure analysis: The example of RNA polymerase II subunits Rpo4/7

Anahit Torosyan[1#], Thomas Wiegand[1#], Maarten Schledorn[1#], Daniel Klose[1], Peter Güntert[1,2,3], Anja Böckmann[4*], Beat H. Meier[1*]

[1]Physical Chemistry, ETH Zurich, 8093 Zurich, Switzerland; [2]Institute of Biophysical Chemistry, Center for Biomolecular Magnetic Resonance, Goethe University Frankfurt, 60438 Frankfurt am Main, Germany; [3]Department of Chemistry, Tokyo Metropolitan University, 1-1 Minami Osawa, Hachioji, Tokyo 192-0397, Japan; [4]Institut de Biologie et Chimie des Protéines, MMSB, Labex Ecofect, UMR 5086 CNRS, Université de Lyon, 7 passage du Vercors, 69367 Lyon, France

# These authors contributed equally

* Correspondence:
Beat H. Meier
beat.meier@nmr.phys.chem.ethz.ch
Anja Böckmann
anja.bockmann@gmx.net

Supporting information

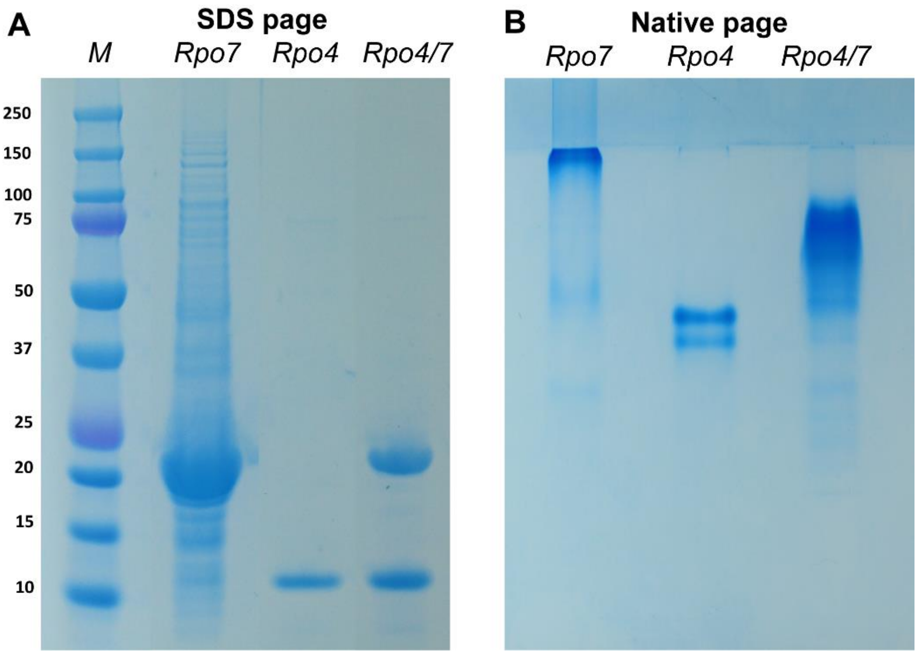

Figure S1. (A) SDS page of Rpo7 (21.2 kDa), Rpo4 (12.3 kDa) and Rpo4/7 complex (33.5 kDa). **(B)** Native page of Rpo7 (theoretical pI 5.2), Rpo4 (theoretical pI 4.7) and Rpo4/7 (theoretical pI 5.0). Note that Rpo7 is barely stable in the absence of Urea or Rpo4 and hence mostly precipitates. Rpo4 contains a contaminant, possibly residual GST tag, which is not present in the Rpo4/7 complex after dimerization (final sample).

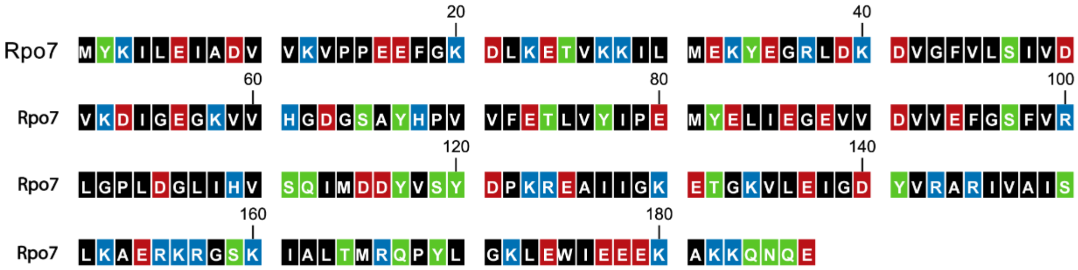

Amino acid composition:

Ala (A)	8	4.3%	Gly (G)	16	8.6%	Pro (P)	7	3.7%
Arg (R)	8	4.3%	His (H)	3	1.6%	Ser (S)	7	3.7%
Asn (N)	1	0.5%	Ile (I)	16	8.6%	Thr (T)	4	2.1%
Asp (D)	13	7.0%	Leu (L)	15	8.0%	Trp (W)	1	0.5%
Cys (C)	0	0.0%	Lys (K)	20	10.7%	Tyr (Y)	9	4.8%
Gln (Q)	4	2.1%	Met (M)	5	2.7%	Val (V)	23	12.3%
Glu (E)	22	11.8%	Phe (F)	5	2.7%			

Figure S2. The primary structure and amino acid composition of Rpo7. Negatively charged amino-acid residues are shown in red, positively charged ones in blue, polar residues in green and hydrophobic ones in black.

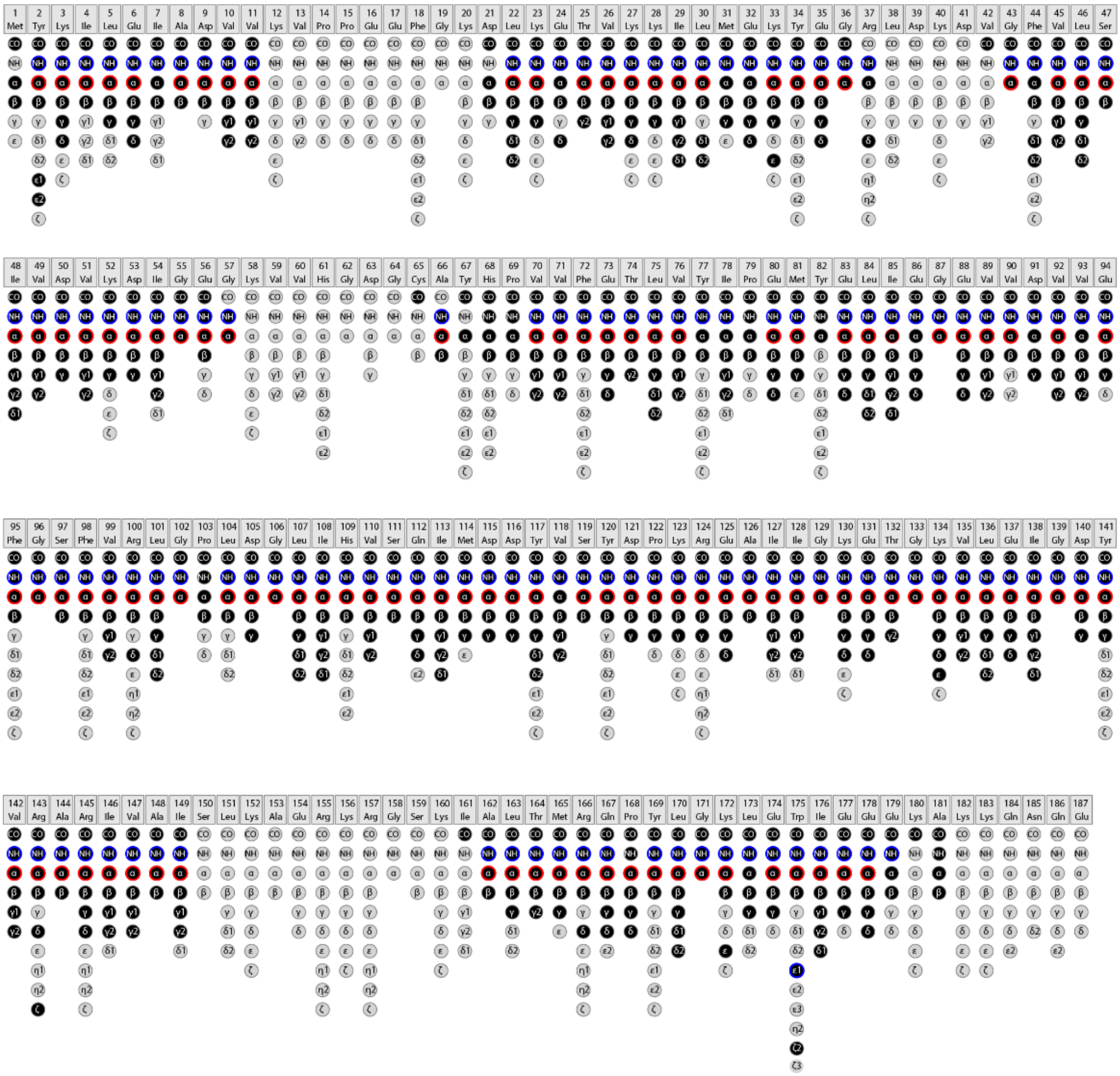

Figure S3. Assignment graph of Rpo4/7* created using the CcpNmr software. Black dots represent assigned spins, grey dots unassigned spins. Amide nitrogens and α-carbons for which directly bound protons have been assigned, are highlighted in blue and red respectively.

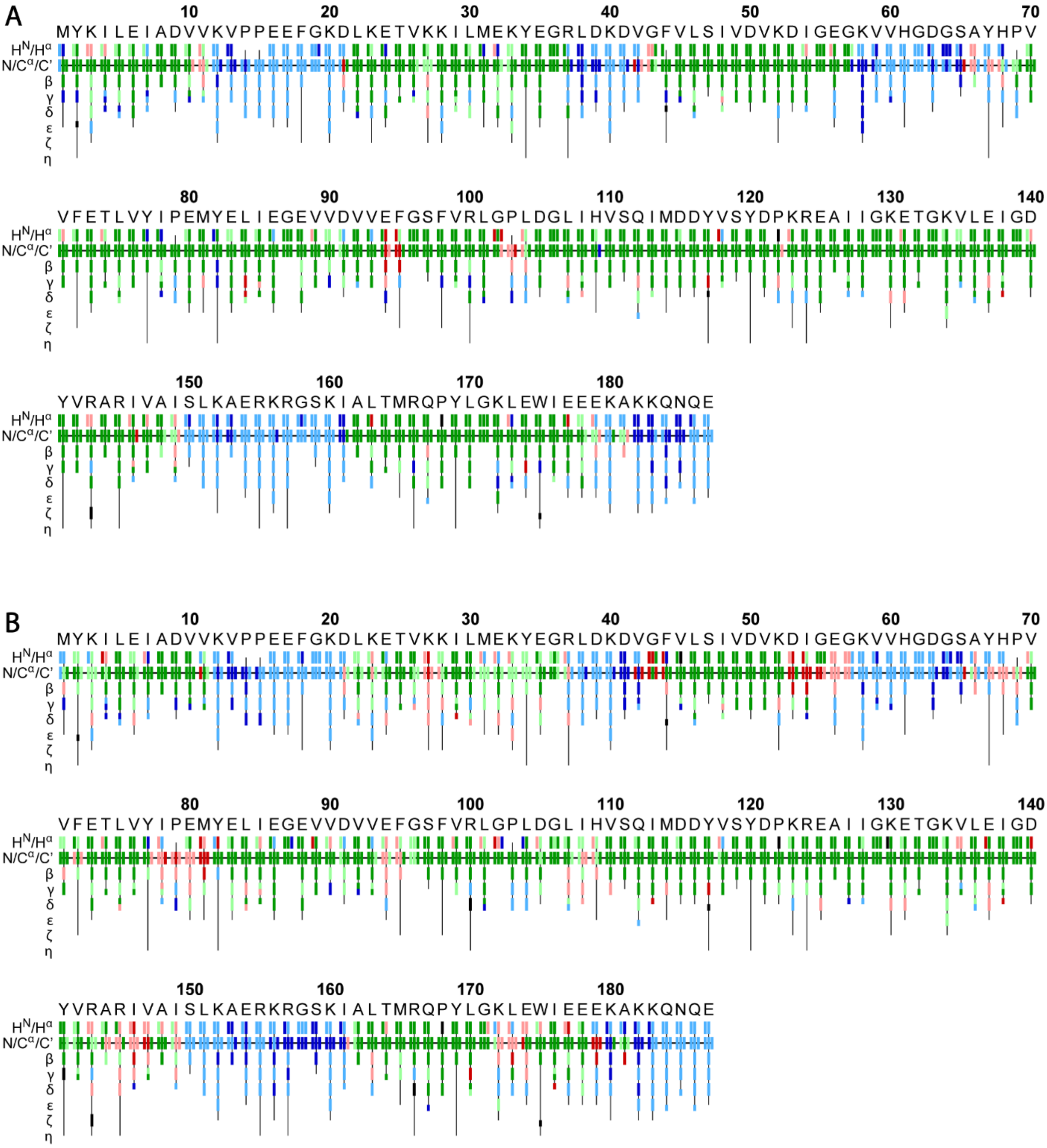

Figure S4. (**A**). Automated resonance assignment by ssFLYA using the manually created peak lists (which resulted from the sequential assignment) extracted from NCACB, CANCO, NCOCX, NCACO, CCC, CANcoCA, NcoCACB, hNCAH, hCANH and hCONH spectra. (**B)** Automated peak assignment by ssFLYA using automated peak lists.

Green: FLYA result agrees with manually assigned values (within tolerance); red: FLYA result does not agree with manually assigned values; blue: assigned by FLYA but no manually assigned values available; black: with reference assignment, but no FLYA assignment available. Dark/light colors: FLYA assignments classified as “strong” or “weak”.

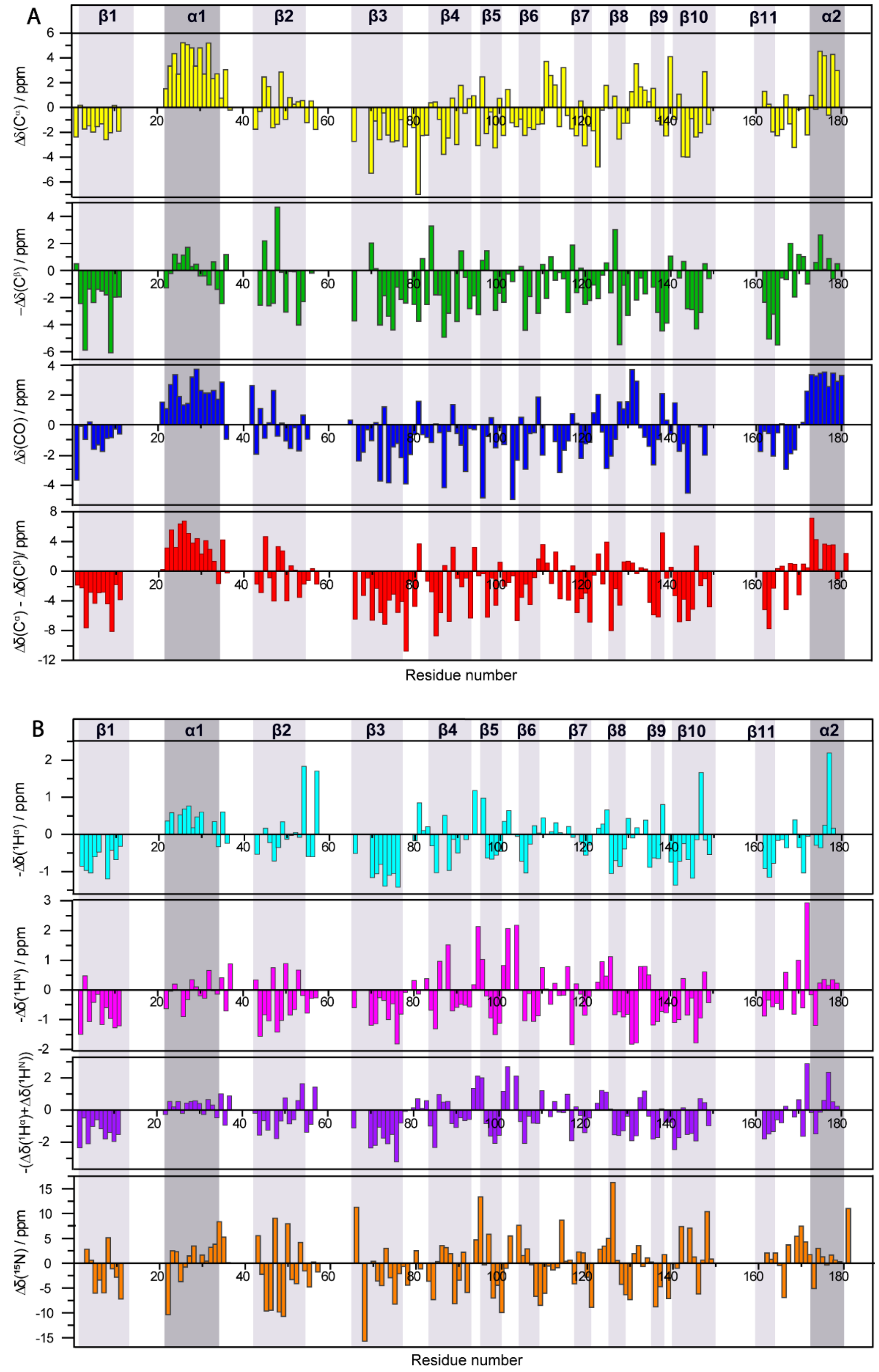

Figure S5 (A): Secondary chemical shifts $\Delta\delta(^{13}C^{\alpha})$ (yellow), $\Delta\delta(^{13}C^{\beta})$ (green), $\Delta\delta(^{13}CO)$ (blue) and difference of $\Delta\delta(^{13}C^{\alpha})$ and $\Delta\delta(^{13}C^{\beta})$ (red) (SCS obtained by subtracting the random-coil shifts from the observed chemical shifts). (**B**): Secondary $^{1}H^{\alpha}$ (cyan), $^{1}H^{N}$ (magenta), the sum of $^{1}H^{\alpha}$ and $^{1}H^{N}$ (purple) chemical shifts (obtained by subtracting the random-coil shifts from the observed chemical shifts), and ^{15}N (orange) SCS. Positive SCS differences indicate α-helices, negative SCS differences β-sheets. Secondary structure elements observed by crystallography are shown as dark (α-helix) and light (β-sheet) grey shaded areas, according to PDB 1GO3.

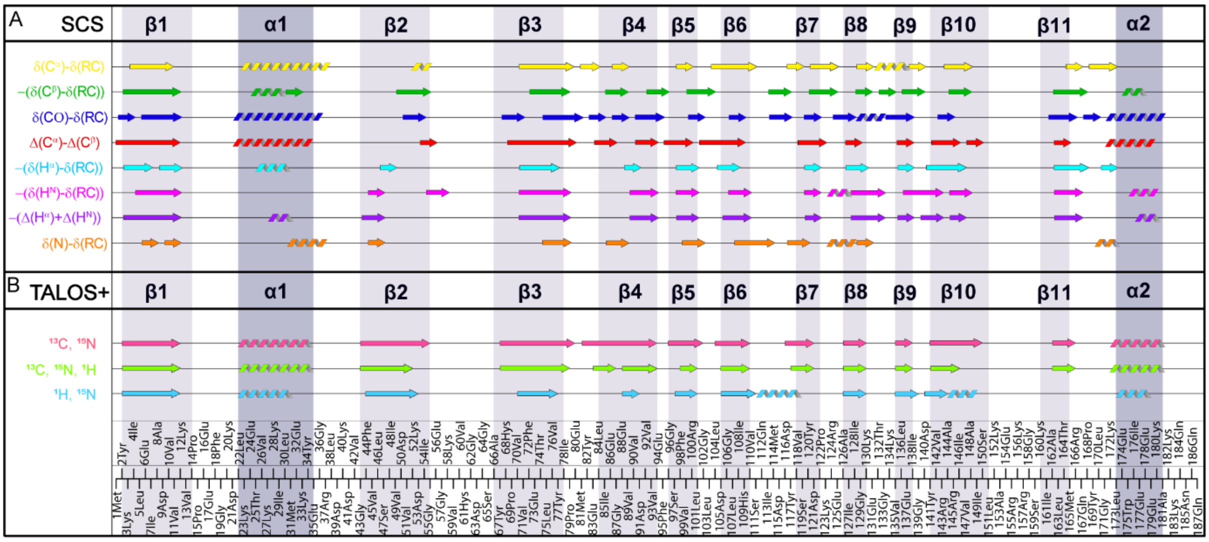

Figure S6 (**A**): Secondary structure based on $^{13}C^{\alpha}$ (red), $^{13}C^{\beta}$ (green), ^{13}CO (blue), difference of $^{13}C^{\alpha}$ and $^{13}C^{\beta}$ (yellow) chemical shifts, $^{1}H^{\alpha}$ (cyan), $^{1}H^{N}$(pink), sum of $^{1}H^{\alpha}$ and $^{1}H^{N}$ chemical shifts (purple) and ^{15}N (orange) chemical shifts obtained by subtracting the random-coil shifts from the observed chemical shifts. (**B**): Secondary structure based on ^{13}C and ^{15}N (light red), ^{1}H and ^{15}N (light blue) and all (light green) chemical shifts using TALOS+ (Shen et al.). Secondary structure elements observed by X-ray crystallography are shown as dark (α-helix) and light (β-sheet) grey shaded areas, according to PDB 1GO3 (ref).

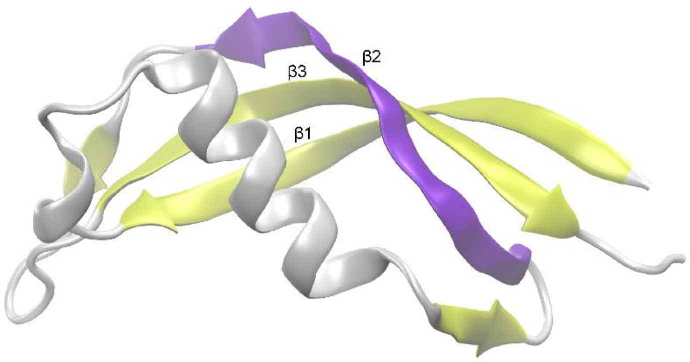

Figure S7. X-ray crystal structure of Rpo7 (PDB 1GO3) for residues 1 to 80. β2 strand is shown in violet, all other β strands are in yellow, remaining part of the protein is in grey.

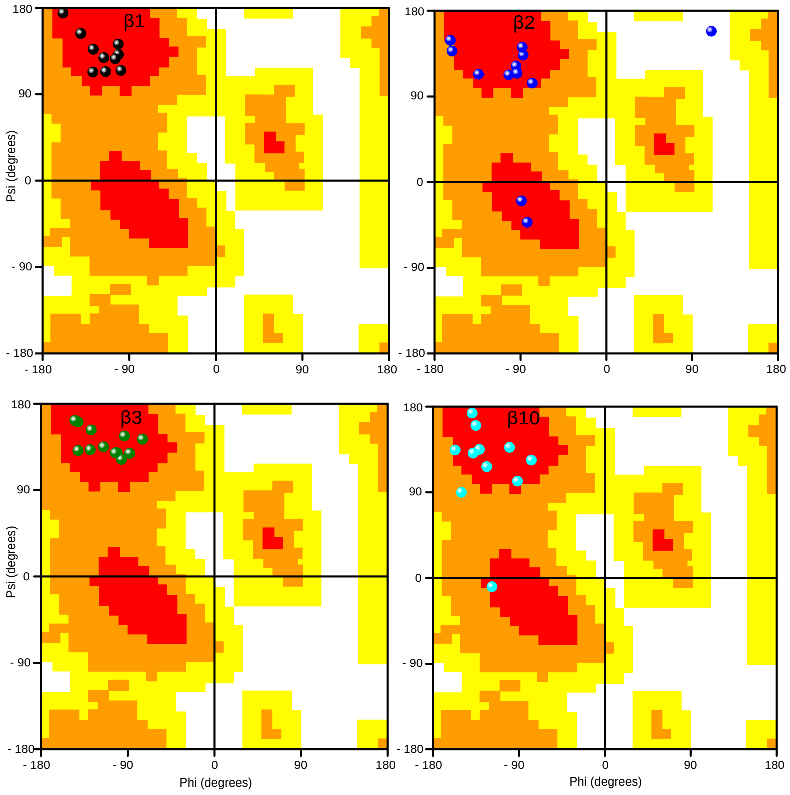

Figure S8. Ramachandran plot for the backbone angles within the four long β-strands β1, β2, β3 and β10 in Rpo7 as defined in Figure 4 (from PDB 1GO3).

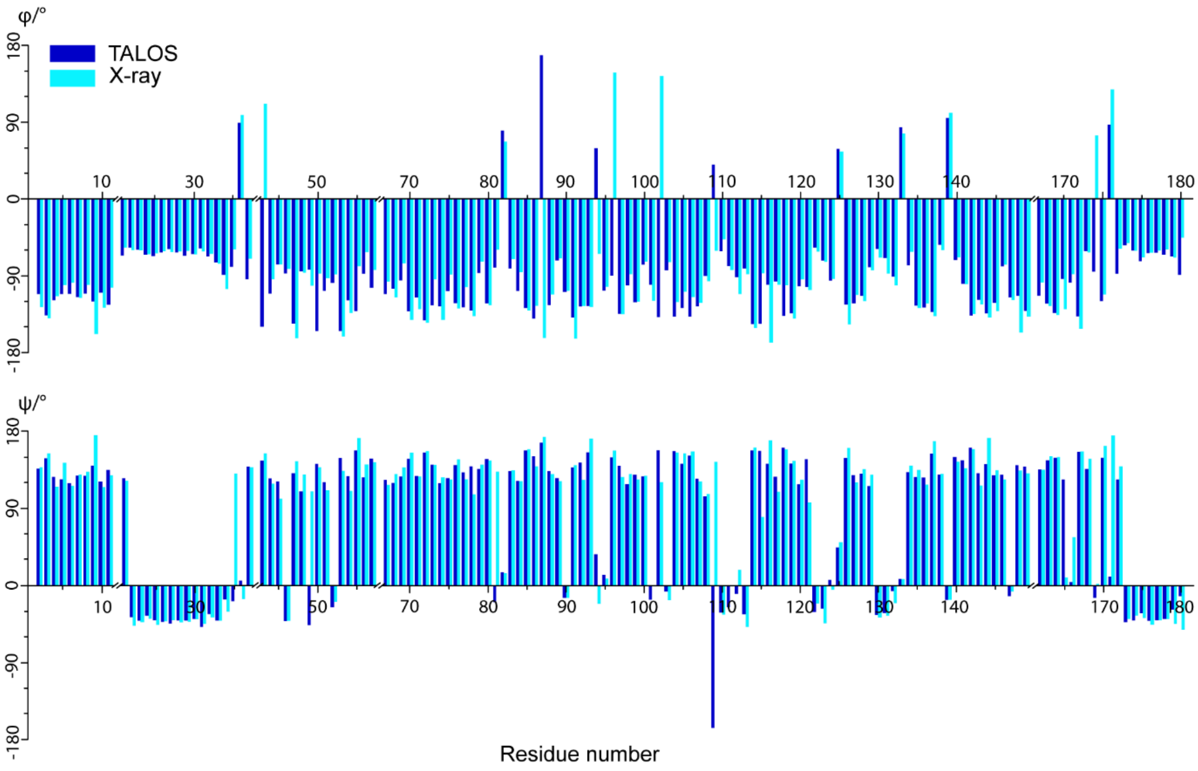

Figure S9. Comparison of backbone dihedral angles Phi (φ) and Psi (ψ) derived from the crystal structure (light blue) and from TALOS+ (dark blue) using the combination of ^{13}C, ^{1}H and ^{15}N data. All residues are shown in the figure for which both crystallographic and TALOS+ data are available.

Table S1. Overview of experimental parameters of the MAS solid-state NMR experiments

**Experiment**	**DARR**	**NCA**	**NCACB**	**NCACX**	**NCOCX**
MAS frequency/kHz	17.0	17.0	17.0	17.0	17.0
Field/T	20.0	20.0	20.0	20.0	20.0
t_1 increments	2560	1536	80	76	68
Sweep width (t1)/kHz	468	774	55	55	55
Acquisition time (t1)/ms	12.8	11.5	8.4	8	7.2
t2 increments	3072	3072	128	132	118
Sweep width (t2)/kHz	468	468	35	35	30
Acquisition time (t2)/ms	15.4	15.4	8.6	8.8	9.2
t3 increments	-	-	2304	2304	2304
Sweep width (t3)/kHz	-	-	468	468	468
Acquisition time (t3)/ms	-	-	11.5	11.5	11.5
^{1}H Spinal64 (Fung et al. 2000) decoupling power/kHz	90	90	90	90	90
Interscan delay/s	2.7	3	3	3	3.1
Number of scans	12	12	8	8	16
Measurement time/h	23:26	17:44	69:00	69:24	114:48
**Transfer I**	**HC-CP**	**HN-CP**	**HN-CP**	**HN-CP**	**HN-CP**
^{1}H field/kHz	60	60	60	60	60
X field/kHz	40.4	44	44	44	43.9
Shape	Tangent ^{1}H	Tangent ^{1}H	Tangent ^{1}H	Tangent ^{1}H	Tangent ^{1}H
^{13}C carrier/ppm	102.8	-	-		
Time/ms	0.7	0.9	0.9	0.9	0.9
**Transfer II**	**DARR**	**NC-CP**	**NC-CP**	**NC-CP**	**NC-CP**
^{1}H field/kHz	17	-	-	-	-
^{13}C field/kHz	-	6	6	6	6
^{15}N field/kHz	-	9.9	10.2	20.7	10.2
Shape	-	Tangent ^{13}C	Tangent ^{13}C	Tangent ^{13}C	Tangent ^{13}C
Carrier/ppm	102.8	58.3	58.3	58.3	176.7
Time/ms	20	6.5	6.5	6.5	5.5
**Transfer III**	**-**	**-**	**DREAM**	**DARR**	**DARR**
^{1}H field/kHz	-	-	-	17	17
^{13}C field/kHz	-	-	7.3	-	-
^{15}N field/kHz	-	-	-	-	-
Shape	-	-	Tangent ^{13}C	-	-
Carrier/ppm	-	-	56	176.7	176.7
Time/ms	-	-	2	80	90
**Experiment**	**CANCO**	**NcoCACB**	**CANcoCA**	**CCC**	
MAS frequency/kHz	17.0	17.0	17.0	17.0	
Field/T	20.0	20.0	20.0	20.0	
t1 increments	132	58	114	142	
Sweep width (t1)/kHz	35	55	35	66	
Acquisition time (t1)/ms	8.8	6.1	7.6	5	
t2 increments	64	118	54	142	
Sweep width (t2)/kHz	55	35	55	66	
Acquisition time (t2)/ms	6.8	7.9	5.7	5	
t3 increments	2304	2304	2304	3072	
Sweep width (t3)/kHz	468	468	468	468	
Acquisition time (t3)/ms	11.5	11.5	11.5	15.4	
^{1}H Spinal64 (Fung et al. 2000) decoupling power/kHz	90	90	90	90	
Interscan delay/s	3	3.2	3.2	3	
Number of scans	8	16	16	8	
Measurement time/h	56:30	97:30	88:40	139:30	
**Transfer I**	**HC-CP**	**HN-CP**	**HC-CP**	**HC-CP**	

^{1}H field/kHz	60	60	60	60	
X field/kHz	42	43.4	60.3	39.1	
Shape	Tangent ^{1}H	Tangent ^{1}H	Tangent ^{1}H	Tangent ^{1}H	
^{13}C carrier/ppm	58.3	-	58.3	56.5	
Time/ms	0.9	0.9	0.7	0.5	
**Transfer II**	**CN-CP**	**NC-CP**	**CN-CP**	**DREAM**	
^{1}H field/kHz	-	-	-	-	
^{13}C field/kHz	6	6	8.6	7.4	
^{15}N field/kHz	10.2	20.7	10.1	-	
Shape	Tangent ^{13}C	Tangent ^{13}C	Tangent ^{13}C	Tangent ^{13}C	
Carrier/ppm	58.3	176.7	58.3	58.1	
Time/ms	6.5	5.5	7	2	
**Transfer III**	**NC-CP**	**Mod. band-selective CP**	**NC-CP**	**DARR**	
^{1}H field/kHz	-	-	-	17	
^{13}C field/kHz	6	11.9	8.6	-	
^{15}N field/kHz	176.7	-	20.7	-	
Shape	Tangent ^{13}C	Tangent ^{13}C	Tangent ^{13}C	-	
Carrier/ppm	176.7	176.7	176.7	42	
Time/ms	5.5	6.5	5.5	60	
**Transfer IV**	**-**	**DREAM**	**Mod. band-selective CP**	**-**	
^{1}H field/kHz	-	-	-	-	
^{13}C field/kHz	-	10.5	11.9	-	
^{15}N field/kHz	-	-	-	-	
Shape	-	Tangent ^{13}C	Tangent ^{13}C	-	
Carrier/ppm	-	58.3	176.7	-	
Time/ms	-	2	6.5	-	
**Experiment**	**hNH**	**hCH**	**hCANH**	**hNCAH**	**hCONH**
MAS frequency/kHz	110	110	110	110	110
Field/T	20	20	20	20	20
t1 increments	788	1624	142	32	58
Sweep width (t1)/ppm	180	200	55	32	20
Acquisition time (t1)/ms	25	19	6	5.8	6.8
t2 increments	5550	2048	32	74	38
Sweep width (t2)/ppm	47	47	32	30	34
Acquisition time (t2)/ms	70	26	5.8	5.8	6.5
t3 increments	-	-	2048	3072	3072
Sweep width (t3)/kHz	-	-	47	47	47
Acquisition time (t3)/ms	-	-	26	38.7	38.7
Proton decoupling (swfTPPM) / kHz	10	10	10	10	10
Nitrogen decoupling (WALTZ64) / kHz	5	5	5	5	5
Water suppression (120 ms MISS.) / kHz	20	20	20	20	20
Interscan delay/s	1	1.2	1	1.1	1
Number of scans	64	32	56	48	88
Measurement time / hh:mm	16:48	19:50	82:58	40:00	64:00
**Transfer I**	**HN-CP**	**HC-CP**	**HC-CP**	**HN-CP**	**HC-CP**
^{1}H field/kHz	89	89	89	89	89
X field/kHz	15	15	15	15	15
Shape	Tangent ^{1}H	Tangent ^{1}H	Tangent ^{1}H	Tangent ^{1}H	Tangent ^{1}H
Carrier/ppm	117.5	56	56	117.5	175
Time/ms	1.6	0.9	0.6	1.5	1.3
**Transfer II**	**NH-CP**	**CH-CP**	**CN-CP**	**NC-CP**	**CN-CP**
^{1}H field/kHz	89	86	-	-	-
^{13}C field/kHz	-	15	71	69	69
^{15}N field/kHz	15	-	35	35	35

Shape	Tangent ^{1}H	Tangent ^{1}H	Tangent ^{13}C	Tangent ^{13}C	Tangent ^{13}C
Carrier/ppm	4.8	4.8	117.5	56	117.5
Time/ms	1	1	16.5	17	16
**Transfer III**	-	-	**NH-CP**	**CH-CP**	**NH-CP**
^{1}H field/kHz	-	-	89	85	89
^{13}C field/kHz	-	-	-	15	-
^{15}N field/kHz	-	-	15	-	15
Shape	-	-	Tangent ^{1}H	Tangent ^{1}H	Tangent ^{1}H
Carrier/ppm	-	-	4.8	4.8	4.8
Time/ms	-	-	1.05	0.55	1.25

Table S2. Statistics of the manually performed peak assignment (data from the CcpNmr software).

Category	Assigned/%	Category	Assigned/%
C	78.6	Gly	75.0
CA	78.1	His	66.7
CB	76.6	Ile	100.0
CG	62.1	Leu	86.7
CD	42.1	Lys	55.0
CE	9.1	Met	100.0
CZ	8.3	Phe	80.0
N	77.0	Glu	81.8
H	75.4	Pro	71.4
HA	67.0	Ser	71.4
Ala	87.5	Thr	100.0
Arg	75.0	Trp	100.0
Asn	0	Tyr	100.0
Asp	76.9	Val	87.0
Gln	50.0		
Cys	0		

Table S3. α-Helices determined from the secondary chemical shifts and from the 3D atomic coordinates deposited in the PDB entry 1GO3 (Todone et al., 2001) using the DSSP algorithm (Kabsch and Sander, 1983).

α-helices and β-sheets	SCS ^{13}C$^{\alpha}$ - ^{3}C$^{\beta}$	SCS ^{1}H$^{\alpha}$ + ^{1}H^N	TALOS+ (^{13}C, ^{15}N)	DSSP
**α1**	Asp21 - Lys33	Lys27 - Leu30	Leu22 - Tyr34	Leu22 - Tyr34
**α2**	Gly171 - Glu178	Ile176 - Glu179	Lys172 - Lys180	Leu173 - Lys180
**3_{10} helix1**	-	-	-	Pro15 - Glu17
**3_{10} helix2**	His109 - Ser111	-	-	Val110 - Gln112
**β1**	Met1 - Val11	Tyr2 - Val11	Tyr2 - Val11	Tyr2 - Val13
**β2**	Asp53 - Gly55	Gly43 - Leu46	Gly43 – Ile54	Gly43 - Ile54
**β3**	His68 - Glu80	Val70 - Ile78	Tyr67 - Pro79	Ala66 - Tyr77
**β4**	Glu83 - Glu86<br>Val90 - Val93	Val89 - val93	Met81 - Val93	Leu84 - Val93
**β5**	Phe95 - Val99	Ser97 - Arg100	Gly96 - Arg100	Gly96 - Arg100
**β6**	Leu101 - Ile108	Asp105 - His109	Leu104 - His109	Asp105 - His109

**β7**	Val118 - Pro122	Ser119 - Asp121	Asp116 - Tyr120	Val118 - Tyr121
**β8**	Ala126 - Ile128	Ile127 - Thr132	Ala126 - Gly129	Ile126 - Gly129
**β9**	Val135 – Glu137	Val135 -Glu137	Val135 - Glu137	Val135 – Leu137
**β10**	Tyr141 – Arg145<br>Val147 - Ile149	Gly139 – Val142<br>Ala144 - Ile146	Tyr141 - Ile149	Tyr141 – Ser150
**β11**	Ala162 – Thr164	Ala162 - Arg166	Ala162 - Met165	Lys160 – Thr164
